# Reduced Fermi Level Pinning at Physisorptive Sites of Moire-MoS_2_/Metal Schottky Barriers

**DOI:** 10.1021/acsami.1c23918

**Published:** 2022-02-28

**Authors:** Zhaofu Zhang, Yuzheng Guo, John Robertson

**Affiliations:** † Department of Engineering, University of Cambridge, Cambridge CB3 0FA, U.K.; ‡ School of Electrical Engineering, Wuhan University, Wuhan 430072, China

**Keywords:** metal contacts, physisorptive sites, MoS_2_, Fermi level pinning, Schottky barriers

## Abstract

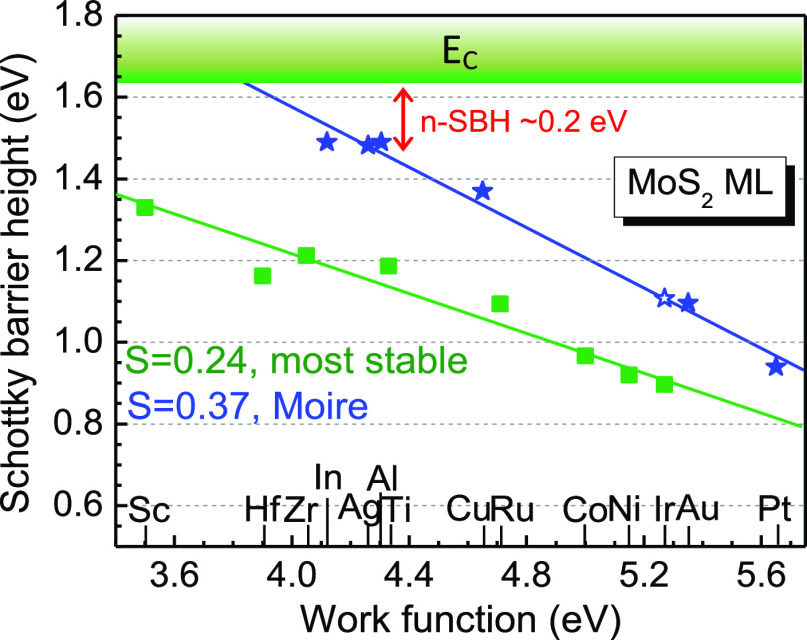

Weaker Fermi level pinning (FLP) at the Schottky barriers of 2D semiconductors is electrically desirable as this would allow a minimizing of contact resistances, which presently limit device performances. Existing contacts on MoS_2_ have a strong FLP with a small pinning factor of only ∼0.1. Here, we show that Moire interfaces can stabilize physisorptive sites at the Schottky barriers with a much weaker interaction without significantly lengthening the bonds. This increases the pinning factor up to ∼0.37 and greatly reduces the n-type Schottky barrier height to ∼0.2 eV for certain metals such as In and Ag, which can have physisorptive sites. This then accounts for the low contact resistance of these metals as seen experimentally. Such physisorptive interfaces can be extended to similar systems to better control SBHs in highly scaled 2D devices.

## 1 Introduction

As transistor downscaling continues, the contact resistances at metal–semiconductor interfaces become an increasing limitation.^1−9^ These resistances arise from the tunnelling or excitation of carriers across the Schottky barriers at these interfaces. At typical interfaces of 2D or 3D semiconductors, the Schottky barrier heights (SBH) are constrained by Fermi level pinning (FLP) due to the metal-induced gap states (MIGS) and similar states.^7−11^ Avoiding FLP would be possible if it were possible to somehow vary the slope factor (or inverse pinning strength) *S* = ∂φ/∂Φ_M_, where φ is the barrier height, Φ_M_ is the metal work function, and *S* is given by^9^
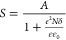
1where *N* is the density of interface gap states, δ is the MIGS decay length into the semiconductor, *e* is the electronic charge, and ε is the interfacial dielectric constant. Increasing *S*, that is, weakening the pinning effect, requires the interface to be more weakly bonded.^6^ A larger *S* (toward the Schottky limit) would allow the SBH to be more easily varied by changing the contact metal work function, which is electrically desirable. However, this is not so easy to arrange.

The interfaces of layered dichalcogenide semiconductors such as MoS_2_ on metals were calculated to have a slope factor of *S* ∼ 0.3 for the defect-free case and an experimental value of *S* ∼ 0.1 for typical defective MoS_2_ contacts.^12−20^ Recently, Liu et al.^6^ achieved a truly weakly bonded metal/MoS_2_ interface and a Schottky-like slope by using a metal film mounted on a polydimethylsiloxane layer, mechanically transferred onto an exfoliated MoS_2_ film, and avoiding any energetic deposition or disordering processes of the metal. They obtained a van der Waals (vdW) interfacial distance of ∼3.0 Å for contacts of noble or precious metals Ag, Cu, Au, and Pt. This achievement of unpinned Schottky barriers is a remarkable demonstration, but this method might be difficult to replicate for realistic devices. Recently, Wang et al.^4^ used In–Au alloys to achieve low-resistance contacts with excellent performance, while Wang et al.^14^ showed by photoemission that Ag has low resistance n-type contacts. Here, we explain why this unusual choice of metals can be expected to have favorable contact properties.

**Figure 1:**
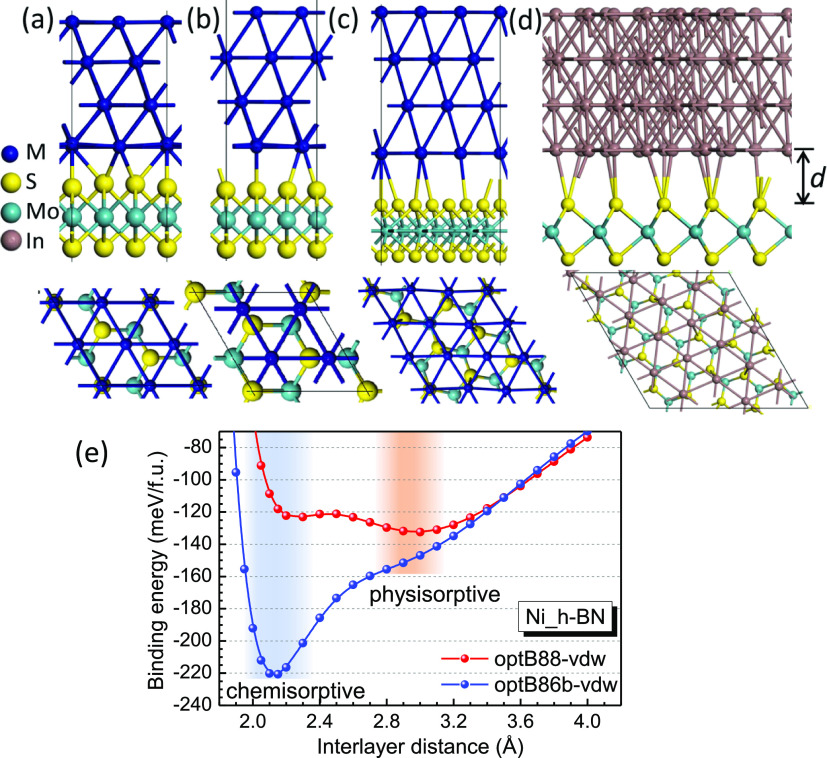
Relaxed Structures of (a) “on-top”, (b) “hollow” chemisorptive sites, and (c) Moire model of physisorptive sites for metals such as Pt, Au, Ir, Ru, Ti, Al, and Ag. (d) Moire MoS_2_(√21 × √21)/In(√19 × √19) model. Note the Moire models in (c,d) have a large interlayer distance (labelled as *d*) and thus weaker interlayer interaction. (e) Calculated interface binding energy for chemisorptive and physisorptive sites of Ni on h-BN with optB88-vdw and optB86b-vdw functionals^32^ for reference, showing a similar trend to ref (21).

The interfacial bonding is generally an intrinsic property of 3D semiconductors and it is not easy to vary. However, for layered 2D semiconductors, unlike the 3D case, a rotational twist of layers as in Moire lattices can be used to favor the more weakly bonded physisorptive interfaces over more strongly bonded chemisorptive interfaces, without significantly lengthening the bonds. This is readily seen for the second-row compound h-BN with lattice-matched metals such as Fe, Co, and Ni. Here, the chemisorptive site is the N on top of the metal site, whereas rotating the h-BN lattice to form the (√3×√3) orientation provides a physisorptive site with both B and N lying off-center and with a much larger interlayer spacing,^21−24^ as schematically shown in Figure 1e. The slope factor of h-BN contacts was found to be dramatically different for each site, *S* ∼ 0.26 for the chemisorptive site, while *S* ∼ 1 for the physisorptive site.^22^

Heavier 2D materials such as MoS_2_ show a similar but less-extreme behavior. The SBH depends mainly on the metal work function^12^ and less on interfacial bond lengths. Computational models of MoS_2_ interfaces found a relatively weak dependence of SBH on bond length.^16^ Nevertheless, an interesting dependence of FLP on bond length was found by Popov et al.^20^ for MoS_2_ interfaces with Ti or Au metals. The Au/MoS_2_ interface was studied in detail by Farmanbar and Brocks^25^ but the SBHs were not studied. Here, we compare the interfacial bond lengths and site geometries of metal/MoS_2_ interfaces and particularly the slope factor *S* and SBHs for a wide range of metals. We are particularly interested in the changes in band alignment and SBHs arising from the changes in interfacial geometries in our idealized models, and less so in the changes in interlayer separation or the impact of any possible defects.

Moire lattices arise from the relative rotation of the lattice axes of each phase, which allows us to vary the interlayer interactions and achieve weakly bonded physisorptive interfaces indirectly without directly lengthening the bond.^25−30^ The interlayer interactions between 2D semiconductors are generally weak vdW bonds. However, interfacial bonds between metals and 2D semiconductors such as MoS_2_ are not just vdW bonds, and the metals can also form multicenter bonds. For heavier 2D semiconductors, different metals can form either chemisorptive or physisorptive bonds with different bonding sites.

## 2 Calculation Methods

The calculations use the plane-wave VASP code^31^ with the GGA functional and PAW pseudopotentials with a cutoff energy of 500 eV. The energy convergence criteria are set to be 10^–5^ eV, while the forces are converged to 0.02 eV/Å. No additional hybrid functional calculation is used to fit the bandgap, as the calculated GGA bandgap of monolayer MoS_2_ is 1.65 eV, close to the experimental optical gap of ∼1.8 eV, and hybrid functionals would cause singularities in the metallic layer. Our key focus is the variation of the SBH slope with metal work function and supercell geometry. The effect of vdW interactions is included using the optB88-vdW functional,^32^ whose chemical trend was preferred to other vdW correction methods,^33^ which are mainly optimized for second row elements.

The calculation involves supercells of the MoS_2_ monolayer and 4 layers of metals, as shown in Figure 1. Note that the Moire pattern is formed in the metal slab and the overall S–Mo–S monolayer. Various metal supercells, some laterally quite large, are expanded from metal (111) (1 × 1) surfaces, with a lattice mismatch under 5%. The supercell lattice constants are set to those of MoS_2_ lattices, while the vertical metal distances are allowed to relax, as metal work functions largely depend on atomic volumes and not lattice constants. A roughly 20 Å vacuum spacing is retained between slabs. We have calculated the interactions between various metal/MoS_2_ interfaces using different superlattices, with work functions ranging from 3.50 eV (Sc) to 5.65 eV (Pt). The supercell lattice-matching conditions, geometries, binding energies, and SBHs are given in Table S1 in the Supporting Information. The metals are given their face-centered cubic (FCC) structures, classified into three groups according to the similarity of lattice constants.

## 3 Results and Discussion

Metals including Pt, Au, Ir, Ru, Ti, Al, and Ag share the same heterostructure models, which consist of three types, as shown in Figure 1. (1) MoS_2_(√3 × √3) axes directly aligned to metal(111) (2 × 2) axes, with the sulfur atoms of MoS_2_ lying at the “on-top” chemisorptive positions of many of the upper metal layer sites, as in Figure 1a. (2) Similar to (1) but with a relative shift in the *xy* plane, with sulfur atoms lying in the “hollow” sites of the upper metal layer, Figure 1b. (3) For physisorptive interfaces, the MoS_2_(√7 × √7) cell axes are rotated to ∼19.1° orientation of the metal(111) (3 × 3) axes to form Moire superlattices, Figure 1c. This choice places most sulfur atoms at physisorptive sites. Note that the Moire lattices can underestimate their actual periodicity for computational purposes.^34^ Other metals such as Ni, Co, and Cu with a smaller lattice constant, or metals such as Zr, Hf, and Sc with larger radii, can also form the interfaces with similar “on-top”, “hollow”, and “Moire” features. Figure 1d shows clearly the bonding in the model of In contacts at Moire interfaces, with the obvious larger interlayer spacing (∼3.2 Å). The full atomic structures of In and Cu interfaces are shown in Figures S1 and S2. Note that the MoS_2_ and metal lattices can also directly align their lattices in (1 × 1) cells but with sizable lattice mismatch, and some of these models are studied for comparison. As a comparison, h-BN shows different adsorption features so that for h-BN, most metals can show physisorptive or chemisorptive sites for each metal, whereas most metals with MoS_2_ show either chemisorptive or physisorptive character for each metal.

The chemical trends of interfacial energy, interfacial bond length, or SBH are plotted against the metal work function.^35^ The interface binding energy is calculated as
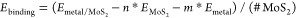
2where *E*_metal/MoS_2__, *E*_metal_, and *E*_MoS_2__ are the total energy of the contact supercell, the metal(111) (1 × 1) surface slab, and the MoS_2_(1 × 1) slab, respectively. #MoS_2_ is the number of MoS_2_ formula units. *n* and *m* are the number of isolated slabs in the heterostructures. A lower *E*_binding_ means a more stable interface.

**Figure 2:**
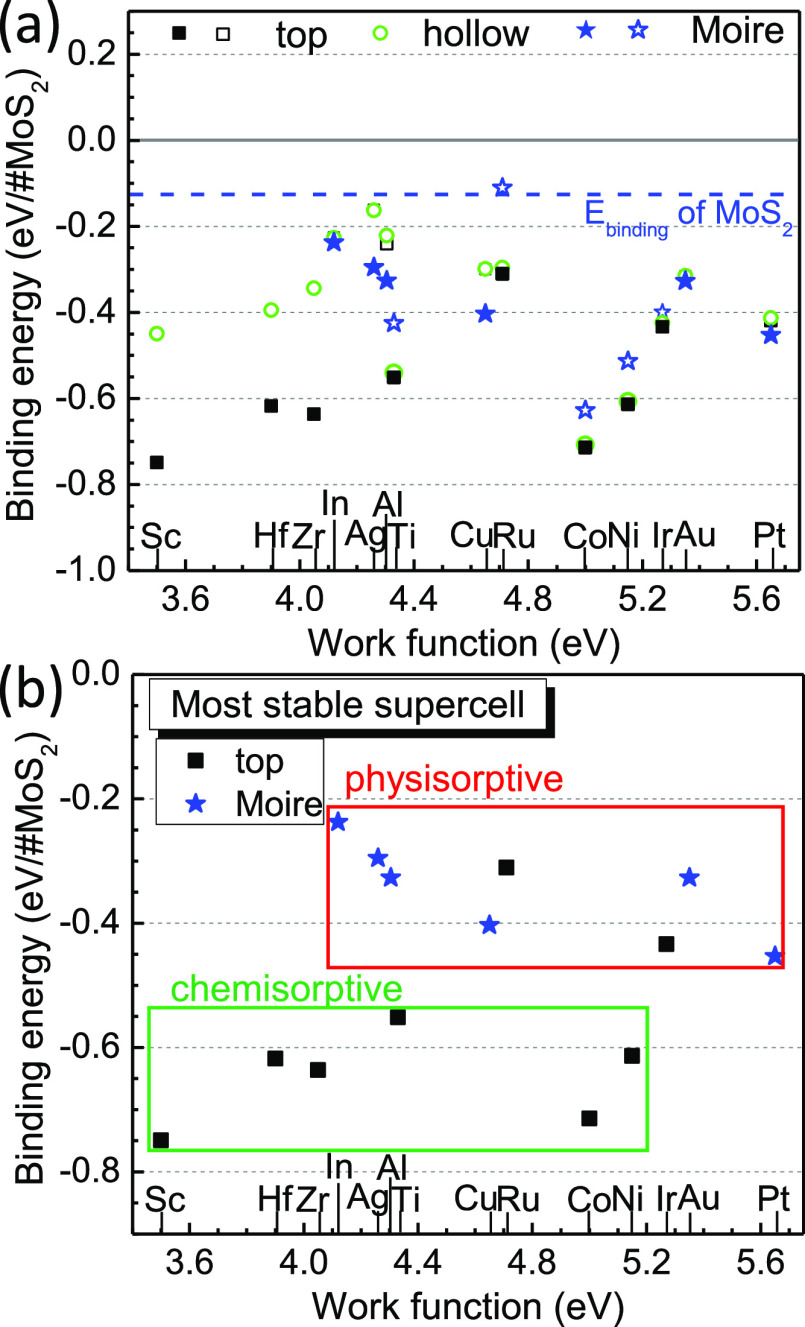
(a) Binding energy for different sites. Filled symbols most stable site, open symbols other sites. The blue line is the binding energy of the MoS_2_ monolayer on another MoS_2_ monolayer, for one surface. (b) Binding energy of the most stable models, classified into the physi- and chemisorptive groups.

The calculated interfacial binding energies for each site geometry are shown in Figure 2a, with the most stable ones are shown again in Figure 2b separated according to their type. These show a stronger binding energy for low work function metals such as Zr–Sc and a slight increase toward medium work function metals, reaching a local maximum around Ru. This is perhaps because the reactive metals such as Ti, Zr, Hf, and so forth form dichalcogenides whose bonding matches that of MoS_2_, whereas the metals of higher work function such as Ru, Co, and Ni have monochalcogenides, while their dichalcogenide phases are endothermic^36^ so their bonding does not match that of MoS_2_. Note that the energy difference between the Moire model and the “on-top” model for the non-noble metals is actually not large.

The most stable configuration of many metals is the on-top site, Figure 1a. For many transition metals, this has a √3 to 2 matching to the MoS_2_ lattice. The metal–MoS_2_ distances shown in Figure 2a vary from the most stable site with close distances for Ni, Co, and Ru to longer but still chemisorptive distances for larger atoms (Sc, Hf, and Zr). Metals such as Ir use this configuration but adopt a longer physisorptive distance. The hollow site geometry is also possible, as in Figure 1b. This has a relatively short chemisorptive bond distance, but there are now three interfacial bonds per sulfur site unlike for the on-top site. Its stability is similar or slightly less than the on-top site in most cases. Note that the metal–semiconducting binding energies can be up to five times larger than those between two semiconducting layers. The most interesting site is the Moire site as in Figure 1c, which mostly forms physisorptive sites. We also studied some large mismatch models with metal(2 × 2)/MoS_2_(2 × 2) matching for comparison. The large mismatch models have much smaller binding energies, thus are not suitable for practical usage because the metal(2 × 2) slabs are directly stacked on MoS_2_(2 × 2) with sizable lattice mismatches, in some cases even reaching 20%. This explains the lower binding energy, even positive values for some cases.

Although there seems to be no obvious trend in Figure 2a, we simplify it by classifying the energy range and interlayer spacing. Figure 2b plots the binding energies of the most stable sites, as classified into chemisorptive or physisorptive groups. The “on-top” site is the most stable site for most non-noble transition metals. This site is classified as a chemisorptive site according to its bond length and binding energy. The Moire site is the most stable for noble metals such as Cu, Ag, Au, and Pt, and their binding energy has a low physisorptive value of numerically below ∼−0.4 eV. For Ir, whose chemical properties are like Pt, the “on-top” site is marginally more stable than the Moire site, but it has a low binding energy comparable to other physisorptive sites. Ru also has a low binding energy, but with the “on-top” configuration.

**Figure 3:**
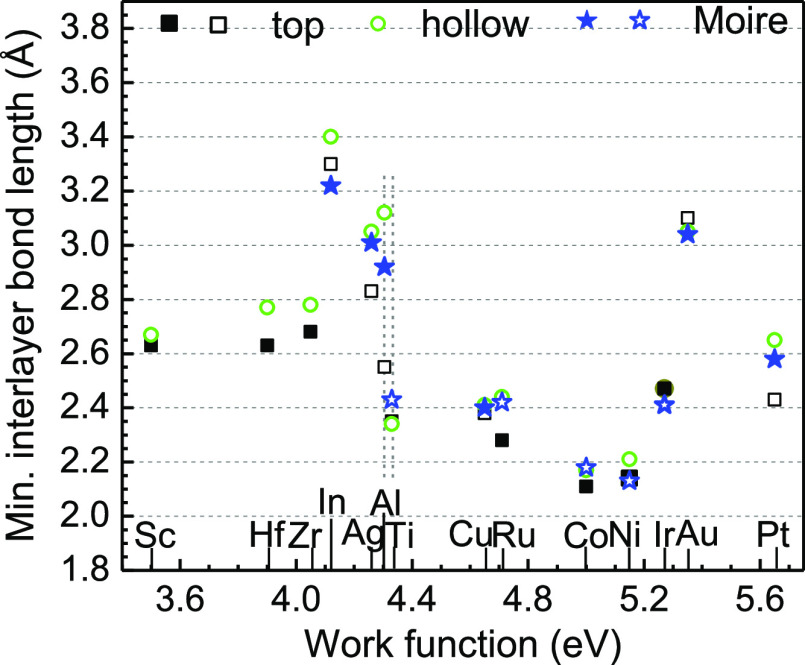
Interlayer bond length diagram. Filled symbols are for the most stable supercells.

**Figure 4:**
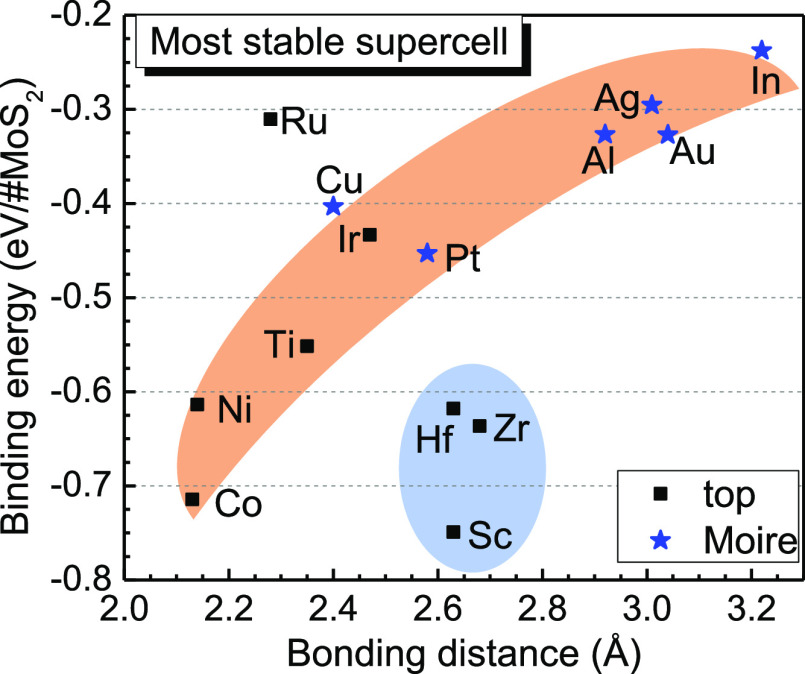
Binding energy vs bonding distance plot. Note that Ru has a shorter bond length but low *E*_binding_. Note that the low work function metals including Sc, Zr, and Hf will react with MoS_2_.

Figure 3 plots the site geometries including the minimum interlayer bond length for each metal. This allows the various bonding types to be classified. Taking Au for example, all three sites of Au have similar bond length and quite weak binding energy and thus they are physisorptive sites. It is interesting to plot the binding energy versus the interfacial bond length as in Figure 4, which extends previous reports.^25^ The interlayer distance and energies are correlated so that a shorter distance generally gives the more negative energy. The long interfacial bond length of In stands out in Figure 3. Note that the low work function metals including Sc, Hf, and Zr are chemically different because they will react with MoS_2_ so that they do not fit the general trend in Figure 4.

**Figure 5:**
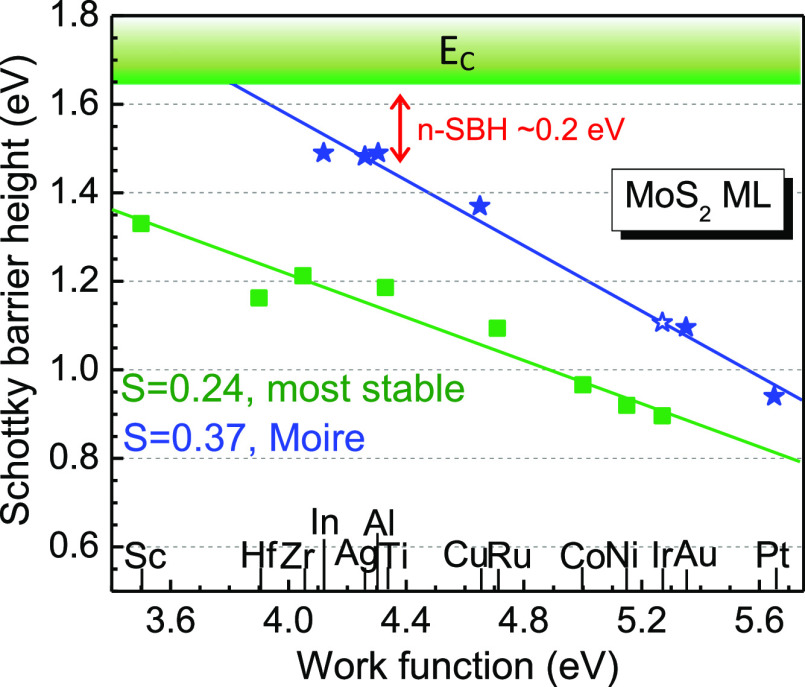
Calculated p-type SBH plot. Filled symbols are for the most stable supercells (“on-top” or Moire). The blue stars are for physisorptive Moire sites. The Fermi level depinning factor from 0.24 to 0.37 is obtained.

Figure 5 plots the p-type SBH for each metal. It is apparent that the chemisorptive and physisorptive configurations in Figure 5 lie on very different *S* slopes. The SBH slope for the stable chemisorptive sites is only *S* = 0.24, the green line in Figure 5, indicating a strong pinning effect. This is consistent with the previous calculations and experimental observations.^12−19^ On the other hand, the SBH value for the physisorptive site in the Moire geometry has a much larger slope of *S* = 0.37, indicating the Fermi level depinning by the physisorptive Moire sites. Note that these Moire sites lead to the overall larger interlayer distance (see Figure 3), that is, the MIGS decay length into the semiconductor in eq 1 is decreased. This also leads to the increased slope *S*. As a result, the n-type SBH is much reduced for some metals such as In at physisorptive Moire sites (∼0.2 eV) compared to the large value of ∼0.5 eV for their on-top configuration, this ensures the experimentally observed low contact resistances.^4^ Although the SBHs show the excellent linear chemical trend versus the contact metal work functions in Figure 5, the trend in binding energy (Figure 2) and interlayer bonding length (Figure 3) is not that obvious. This is because the interface energy and structural trend is more related to other properties such as the metal position in the Periodic table rather than the metal work function.

This Moire lattice ordering also occurs for MoS_2_ deposited on Au.^25,26,34,37^ Bruix et al.^34,37^ studied another larger Moire pattern consisting of a (√13 × √13) R13.9° cell of MoS_2_ on Au(111) (4 × 4), with a similar n-type SBH of ∼0.6 eV to our result. We also studied these R13.9° Moire supercells of Au and Ag, with results shown in Table S1. The small strain and the different S–Au bonding patterns allow such Moire models to represent various experimental situations.^34,37^

Two particular metals with physisorptive sites stand out. The first case is Indium. Indium has a most unusual behavior with a very large physisorptive bond length of ∼3.2 Å and a small binding energy of −0.24 eV at a Moire site, without being a noble metal. Indium also has an unusual on-top configuration but with physisorptive binding energy as shown in Figure 2a. The weak interfacial interaction leads to a small variation of binding energy between different configurations, perhaps due to the metal’s s-like character. The long interlayer bond distance agrees well with lattice images seen by Wang et al.^4^ The weak interaction of In (and Al) with outer S layers of MoS_2_ contrasts with such metals’ strong interaction with oxygen.^38^ The experimental bond lengths^4^ support the vdW parameterizations of Klimeš et al.^32^

The low resistance of In contacts on MoS_2_ (or WS_2_) seen experimentally was attributed to the cleanliness of this contact.^4^ Our results show that this low contact resistance also arises from its larger *S* slope of the physisorptive site of In/MoS_2_, the low work function of In, and thus the closeness of its Fermi level to the MoS_2_ conduction band edge, which is a critical factor in giving its low interface resistance.

The other case is silver. The Fermi energy of Ag contacts lies quite close the conduction band edge of MoS_2_ so the n-SBH is very small (∼0.2 eV) for this geometry, consistent with the small energy difference seen experimentally by photoemission by Wang et al.^14^ Although Ag is a noble metal, its work function is surprisingly low for a noble metal. It is interesting to compare the SBHs of Ag and Au. While Cu, Ag, and Au are all noble metals, Au has a large work function of 5.35 eV as expected for an unreactive metal. However, Ag is noble but has a surprisingly small work function of only 4.25 eV,^35^ similar to that of Ti, but its reactivity with say oxygen is strikingly different.^38^ Despite the difference in work function, they both have a low interfacial binding energy of ∼−0.4 eV for the Moire configuration, with a much larger p-type SBH value than the “on-top” configuration. This guarantees the reduced FLP in such physisorptive Moire-MoS_2_/metal Schottky barriers. Thus, this Moire configuration has the lowest n-SBH for these metals, as seen experimentally.^15^

With these two special cases, we may find that with the noble metals such as Au, Ag, and Cu, or the s-electron metals such as In and Al, it is easier to form the physisorbed Moire pattern at metal/MoS_2_ interfaces. This principally can be used to select the contact metals to achieve such physisorptive sites.

**Figure 6:**
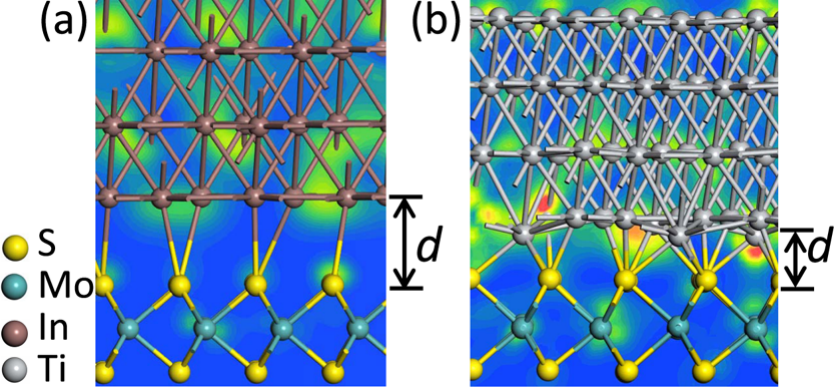
Charge density diagram for (a) In(√19 × √19)/MoS_2_(√21 × √21) and (b) Ti(3 × 3)/MoS_2_(√7 × √7) Moire interfaces. Note the difference in interfacial charge density as the interfacial bond length increases.

It is interesting to compare the behavior of physisorptive and chemisorptive interfaces in terms of the charge density distributions, in Figure 6. It is clear that there is a much reduced valence charge across the interface for the physisorptive configuration at the In/MoS_2_ interface owing to this larger interlayer distance when compared to the reactive Ti/MoS_2_ interface. This may arise from the s-like compared to d-like character of these two contact metals. The d-like Ti has multiple orbital lobes, which allow it to interact for any spacing with Ti, whereas In sites must maintain a certain phase if they were to interact with MoS_2_. It is also seen that the In contact achieves a much flatter interface plane, while the atomic roughness at Ti/MoS_2_ plane is serious, owing to the actively reactive properties of Ti. Besides, the In contact has an obviously larger interlayer spacing (3.12 Å) compared to Ti/MoS_2_ case (2.31 Å), owing to the weak interface interaction.

Recently, Shen et al. reported the low contact resistance between semimetal bismuth and monolayer MoS_2_.^5^ Bismuth is a semimetal that has a very low density of states around E_F_, which provides weak pinning of its SBHs on Bi or similar Sb when contacting to 2D materials^5^ or Si.^39,40^ However, Bi has a relatively lower melting point of only 271 °C, thus Bi contacts will melt during back-end-of-line (BEOL) processing. Sb with a higher melting point of 631 °C is able to survive BEOL temperature.^41^

It is useful to compare the behavior of chemisorptive and physisorptive sites on MoS_2_ and h-BN. Although for h-BN, both types of site can occur for the same metal,^21^ depending on the orientation of the lattices, for MoS_2_ generally a particular chemi- or physisorptive site occurs for different metals as seen in Figure 5 and the effect is only seen when comparing the overall behavior between the two types of interfacial bonds. Compared to MoS_2_, h-BN has very strong B–N electron-pair bonding. This creates a more extreme variation of pinning factor *S* between the chemi- and physisorptive sites for h-BN/metal interfaces, from *S* ∼ 1 to 0.25.^22,23^ On the other hand, for MoS_2_, each sulfur atom with three neighboring Mo atoms already has multicenter bonding rather than electron-pair bonding as in h-BN. Thus, at MoS_2_/metal interfaces, sulfur atoms can easily bond to metal atoms with the multicenter bonding character.

**Figure 7:**
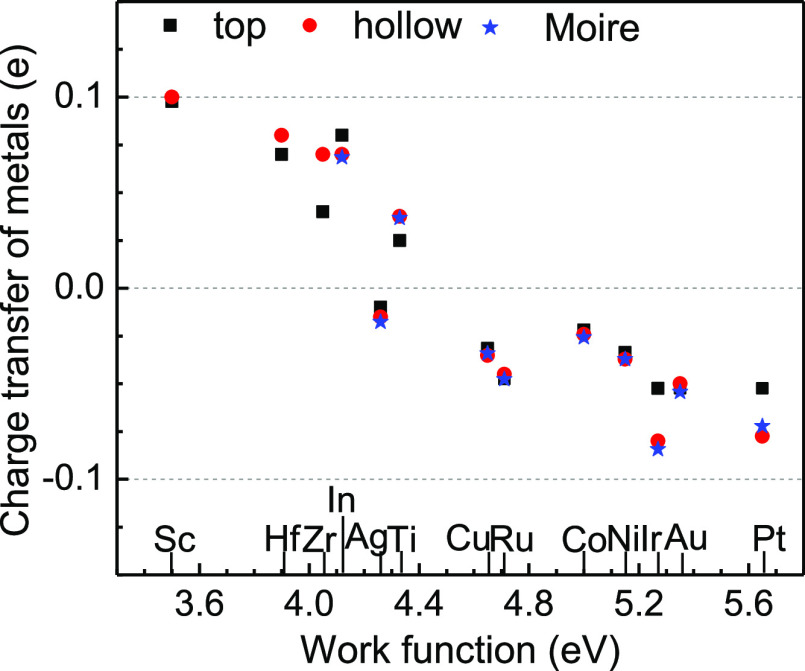
Charge transfer between metal slabs and MoS_2_ per interface metal atoms. The positive value indicates that electrons are transferred from the metals to semiconductor side.

Figure 7 plots the charge transfer between the MoS_2_ layer and the metal slab. It is seen that the metal slabs lose 0.1 electrons per atom for Sc. With higher work function, the charge transfer decreases to around zero at the medium work function near 4.4 eV, where the charge transfer passes through neutral for Cu–Co. Then, electrons are gradually transferred from the semiconductor side back to the metal side of the interface. It reaches −0.06 electrons per atom for Pt. Interestingly, the charge transfer is little dependent on the site symmetry. Ag stands out as having a more negative charge, showing its noble metal character.

## 4 Conclusions

In conclusion, we studied the interfacial bonding and SBHs of MoS_2_ monolayer interfaces with various metal contacts. It is found that the “on-top” chemisorptive site is the most stable configuration for many metals, but the Moire pattern is more favorable to cause noble and other metals such as In and Al to adopt physisorptive sites. These Moire sites are important in having a lower n-type barrier height and their Moire pattern configurations enable greater Fermi level *depinning* than other geometries. The n-SBH is found to be lowest for these sites particularly for Ag, as seen experimentally. The interesting In(Au)/MoS_2_ Moire pattern is consistent with experiments.

## Supporting Information Available

Lattice matching, interlayer spacing, bond length, binding energies, and SBHs for the MoS_2_/metal supercells; the atomic structures of In/MoS_2_ and Cu/MoS_2_ models (PDF)
