# Supplementary material for: Reduced Fermi Level Pinning at Physisorptive Sites of Moire-MoS2/Metal Schottky Barriers
Source: ACS Appl Mater Interfaces. 2022 Feb 28;14(9):11903–9. doi: 10.1021/acsami.1c23918 (PMC9098114; doi:10.1021/acsami.1c23918)
Supplement: Supplementary file 1 — am1c23918_si_001.pdf [file am1c23918_si_001.pdf]

Supporting Information

Reduced Fermi Level Pinning at Physisorptive Sites of Moire-MoS_2/Metal Schottky Barriers

Zhaofu Zhang[†,*], *Yuzheng Guo*[‡], *John Robertson*[†,*]

† Department of Engineering, University of Cambridge, Cambridge CB3 0FA, UK
‡ School of Electrical Engineering, Wuhan University, Wuhan, 430072, China

Corresponding Author
* E-mail: zz389@cam.ac.uk
* E-mail: jr@eng.cam.ac.uk

Table S1 The lattice matching for the MoS_2/metal supercells with 'on-top' (T), 'hollow' (H), and Moire (M) configurations on each metal. The positive mismatch data means that lattices of metal slabs are larger than MoS_2 slab, thus metal is compressed strained, and vice versa. The larger Au(Ag)(4×4)/MoS_2(√13×√13) Moire model with rotation angel of 13.9° by Bruix [34] is also calculated in our work for comparison.

M	Matching method	Mis-match	Interlayer spacing (A)	Min. bond length (A)	$E_{binding}$ (eV/#MoS_2)	SBH (eV)
Pt	MoS2(√3×√3)_Pt(2×2)_T	1.8%	2.43	2.43	-0.419	0.87
	MoS2(√3×√3)_Pt(2×2)_H	1.8%	2.51	2.65	-0.413	0.96
	MoS2(√7×√7)_Pt(3×3)_M19.1°	-0.06%	2.41	2.58	-0.452	0.97
Au	MoS2(√3×√3)_Au(2×2)_T	4.4%	3.11	3.1	-0.315	0.96
	MoS2(√3×√3)_Au(2×2)_H	4.4%	3.01	3.05	-0.315	0.95
	MoS2(√7×√7)_Au(3×3)_M19.1°	2.5%	3.04	3.04	-0.326	1.10
	MoS2(√13×√13)_Au(4×4)_M13.9°	0.3%	2.98	2.99	-0.269	1.20
Ir	MoS2(√3×√3)_ Ir(2×2)_T	-0.8%	2.35	2.47	-0.433	0.90
	MoS2(√3×√3)_ Ir(2×2)_H	-0.8%	2.35	2.47	-0.424	1.05
	MoS2(√7×√7)_ Ir(3×3)_M19.1°	-2.6%	2.24	2.41	-0.399	1.11
Ni	MoS2(3×3)_Ni(4×4)_T	4.1%	2.14	2.14	-0.613	0.92
	MoS2(2×2)_Ni(√7×√7)_H	3.3%	2.11	2.21	-0.612	0.92
	MoS2(2×2)_Ni(√7×√7)_M	3.3%	2.13	2.13	-0.513	0.96
Co	MoS2(3×3)_Co(4×4)_T	4.1%	2.11	2.11	-0.714	0.97
	MoS2(2×2)_Co(√7×√7)_H	3.3%	2.09	2.17	-0.712	0.94
	MoS2(2×2)_Co(√7×√7)_M	3.3%	2.18	2.18	-0.627	0.98
Ru	MoS2(√3×√3)_Ru(2×2)_T	-2.1%	2.28	2.28	-0.310	1.09
	MoS2(√3×√3)_ Ru_(2×2)_H	-2.1%	2.32	2.44	-0.300	0.90
	MoS2(√7×√7)_ Ru(3×3)_M19.1°	-3.9%	2.31	2.42	-0.110	1.18
Cu	MoS2(4×4)_Cu(5×5)_T	0.3%	2.38	2.38	-0.299	1.38
	MoS2(4×4)_Cu(5×5)_H	0.3%	2.32	2.41	-0.300	1.40
	MoS2(√12×√12)_Cu(√19×√19)_M9.5°	1.0%	2.32	2.40	-0.403	1.37
Ti	MoS2(√3×√3)_ Ti(2×2)_T	5.2%	2.12	2.35	-0.550	1.12
	MoS2(√3×√3)_ Ti(2×2)_H	5.2%	1.90	2.34	-0.551	1.10
	MoS2(√7×√7)_ Ti(3×3)_M19.1°	3.3%	2.31	2.43	-0.425	1.11
Al	MoS2(√3×√3)_ Al(2×2)_T	3.3%	2.55	2.55	-0.240	1.33
	MoS2(√3×√3)_ Al(2×2)_H	3.3%	3.01	3.12	-0.221	1.33
	MoS2(√7×√7)_ Al(3×3)_M19.1°	1.5%	2.92	2.92	-0.326	1.49
Ag	MoS2(√3×√3)_ Ag(2×2)_T	4.4%	2.83	2.83	-0.163	1.37
	MoS2(√3×√3)_ Ag(2×2)_H	4.4%	2.92	3.05	-0.161	1.38
	MoS2(√7×√7)_ Ag(3×3)_M19.1°	2.5%	2.88	3.01	-0.295	1.48
	MoS2(√13×√13)_Ag(4×4)_M13.9°	0.4%	2.79	2.80	-0.373	1.52
In	MoS2(2×2)_ In(2×2)_T	6.3%	3.30	3.30	-0.226	1.51
	MoS2(2×2)_ In(2×2)_H	6.3%	3.24	3.70	-0.226	1.46
	MoS2(√21×√21)_In(√19×√19)_M12.5°	1.1%	3.12	3.22	-0.237	1.49
Zr	MoS2(2×2)_Zr(2×2)_T	0.5%	2.68	2.68	-0.636	1.21
	MoS2(2×2)_Zr(2×2)_H	0.5%	2.09	2.78	-0.343	0.99
Hf	MoS2(2×2)_Hf(2×2)_T	-0.5%	2.63	2.63	-0.617	1.16
	MoS2(2×2)_Hf(2×2)_H	-0.5%	2.06	2.76	-0.394	1.04
Sc	MoS2(2×2)_Sc(2×2)_T	2.4%	2.63	2.63	-0.749	1.33
	MoS2(2×2)_Sc(2×2)_H	2.4%	1.97	2.67	-0.449	1.21

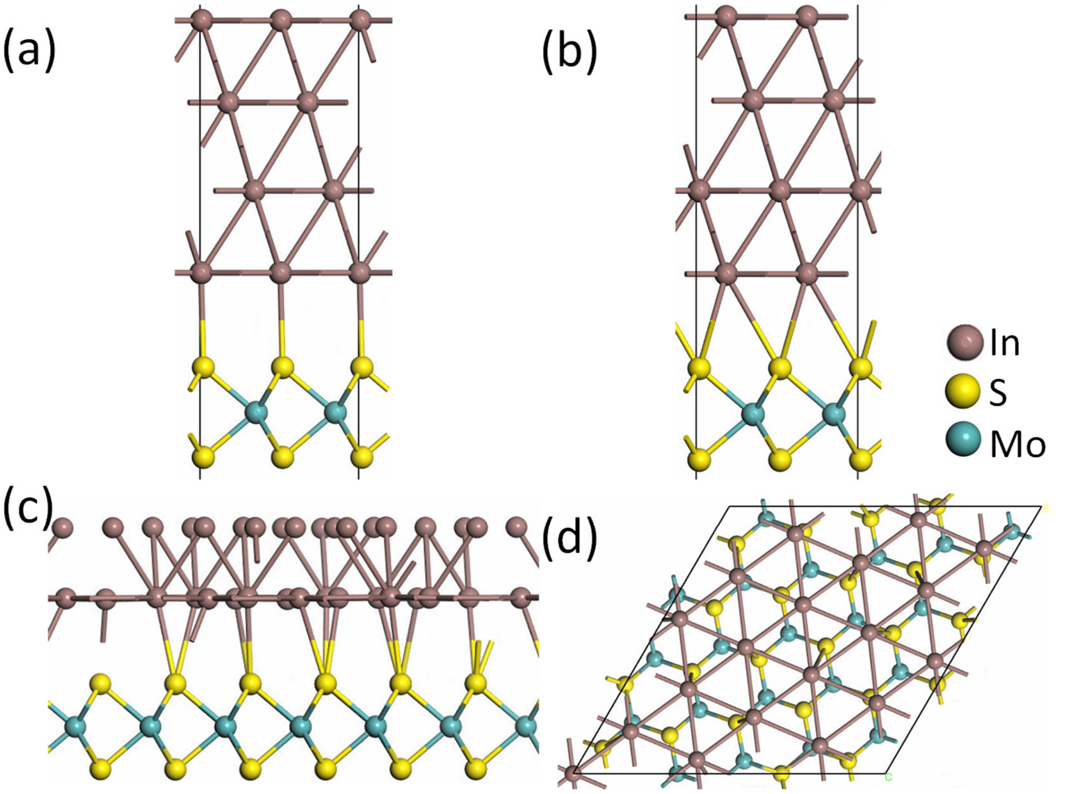

Figure S1. The atomic structures of the (a) ‘top’ model, (b) ‘hollow’ and (c,d) Moire models for In. The Moire model uses $MoS_2(\sqrt{21}\times\sqrt{21})/In(\sqrt{19}\times\sqrt{19})$ matching.

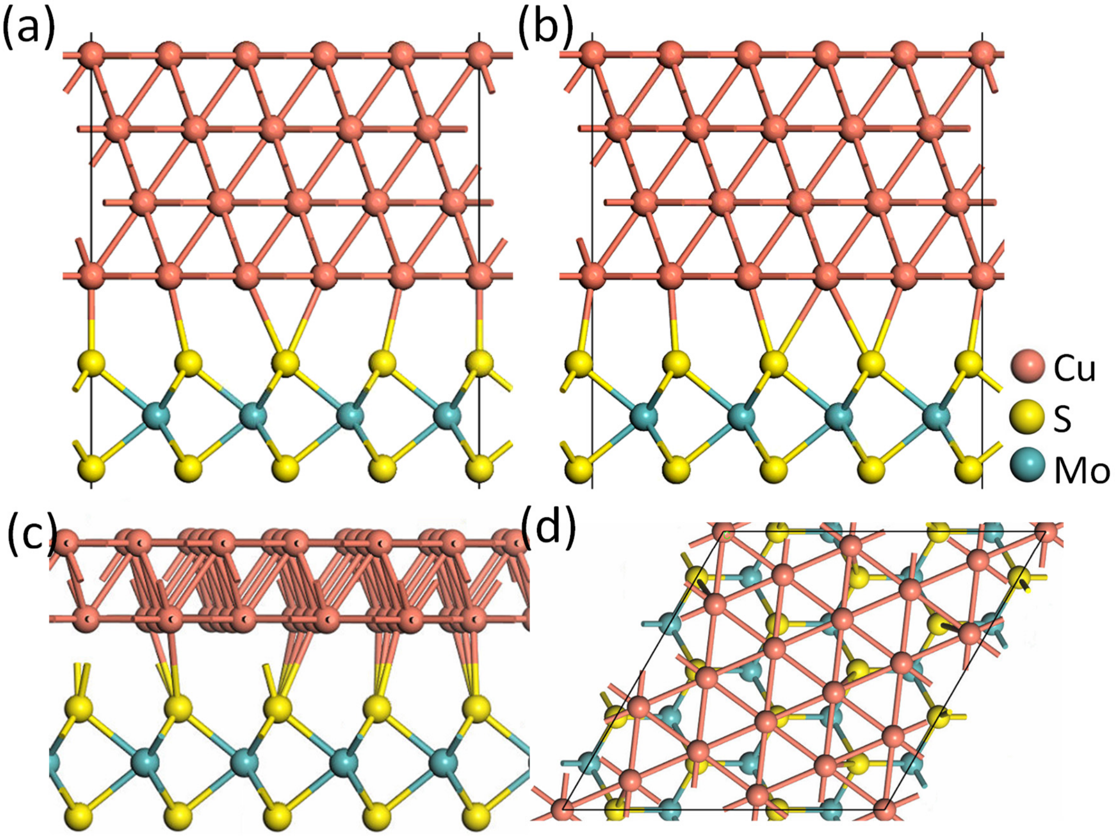

Figure S2. The side and top views of the (a) “top” model, (b) “hollow” and (c,d) Moire model for Cu. Both the (c) side view and (d) top view are given for the Moire model. The Moire model uses $MoS_2(\sqrt{12}\times\sqrt{12})/Cu(\sqrt{19}\times\sqrt{19})$ matching.
